# *Ganoderma lucidum* Extract Reduces the Motility of Breast Cancer Cells Mediated by the RAC–Lamellipodin Axis

**DOI:** 10.3390/nu11051116

**Published:** 2019-05-19

**Authors:** Ariana Acevedo-Díaz, Gabriela Ortiz-Soto, Ivette J. Suárez-Arroyo, Astrid Zayas-Santiago, Michelle M. Martínez Montemayor

**Affiliations:** 1 Department of Biology, University of Puerto Rico at Bayamón, Bayamón, PR 00959, USA; ari.aceve12@gmail.com; 2 Department of Biochemistry, Universidad Central del Caribe-School of Medicine, Bayamón, PR 00960, USA; gabriela.ortiz.soto@gmail.com (G.O.-S.); ivette.suarez@uccaribe.edu (I.J.S.-A.); 3 Department of Pathology and Laboratory Medicine, Universidad Central del Caribe-School of Medicine, Bayamón, PR 00960, USA; astrid.zayas@uccaribe.edu

**Keywords:** breast cancer, cancer cell migration, *Ganoderma lucidum*, lamellipodin, Rac

## Abstract

Breast cancer (BC) is the second leading cause of cancer death among women worldwide. The main cause of BC morbidity and mortality is the invasiveness capacity of cancer cells that may lead to metastasis. Here, we aimed to investigate the therapeutic efficacy of *Ganoderma lucidum* extract (GLE)—a medicinal mushroom with anticancer properties—on BC motility via the Rac/Lamellipodin pathway. GLE treatment effects were tested on MDA-MB-231 breast cancer cells. The effects were tested on cell viability, migration and invasion. Pulldowns, immunoblotting, and immunofluorescence were used to measure Rac activity and the expression of proteins involved in cell migration and in lamellipodia formation, respectively. As a result, GLE suppressed BC cell viability, migration, and invasion capacity. GLE impaired Rac activity, as well as downregulated Lamellipodin, ENA/VASP, p-FAK (Tyr925), Cdc42, and c-Myc expression. Lamellipodia formation was significantly reduced by GLE. In conclusion, we demonstrate that GLE reduces Rac activity and downregulates signaling molecules involved in lamellipodia formation. These novel findings serve as basis for further studies to elucidate the potential of GLE as a therapeutic agent regulating the Rac/Lamellipodin pathway in BC metastasis.

## 1. Introduction

Breast cancer (BC) is the most commonly diagnosed cancer and the second leading cause of cancer death among women worldwide [1,2]. Its high morbidity and mortality are mainly attributed to the high invasive behavior of BC cells, a phenotype that eventually leads them to metastasis. Cancer metastasis comprises numerous interdependent processes, which include uncontrolled growth of cancer cells, invasion to surrounding tissues, migration to distant sites of the body, and colonization of other organs [3]. Among these processes, abnormal regulation of cell migration plays a critical role promoting the dissemination of cancer cells from the primary tumor to other distant tissues [4,5].

Cell migration along the tissues arises from a continuous cycle of synchronized and interdependent steps that involve the re-arrangement of the cytoskeletal machinery [6]. Migratory cells undertake molecular and cellular changes including the remodeling of their cell-cell and cell–matrix adhesion and their actin cytoskeleton which comprises molecular processes involving the activity of various signaling networks [7]. Migration commences when a cell responds to an external signal promoting the polarization and extension of the leading edge in the direction of the movement [6]. The cell cytoskeleton has to be rearranged to shape the leading edge protrusions and generate mechanical forces to retract and detach the cell tail from the extracellular matrix [4].

The Rho GTPases (Rho, Rac, and Cdc42) are migration associated proteins that play a pivotal role in cancer malignancy via the regulation of cell motility, proliferation, apoptosis, gene expression, and invasion [8,9]. In cancer, Rac has been found to be hyperactivated rather than mutated by a deregulation in expression or activity of the upstream regulators known as guanine nucleotide exchange factors (GEF). Additionally, hyperactivation of Rac is due to mutations and impairment of Rac proteosomal degradation in tumors [10]. Rac hyperactivity has been correlated with increased cancer cell survival [11,12]. Moreover, Rac is a strong regulator of actin polymerization and cell surface extensions at the leading edge of motile cells and promotes the formation of lamellipodia through various effectors such as lamellipodin (Lpd).

Lpd transmits signals to the cytoskeleton during cell migration processes promoting actin polymerization by interacting with F-actin and the actin effectors Ena/VASP and the Scar/WAVE complex [13,14,15,16]. Mainly, Ena/VASP proteins promote actin filament elongation and the Scar/WAVE complex regulates actin nucleation through the Arp2/3 complex [16]. The localization of Lpd has been identified in the edges of lamellipodia, the tips of filopodia, and in clathrin-coated pits [15,17]. Lpd is required for lamellipodia formation and maintains the balance of actin nucleation, branching and filament elongation to promote protrusions, and dynamics during cellular response to growth factors. Overexpression of Lpd promotes metastasis in vivo and correlates with reduced metastasis free survival in BC patients [18]. Therefore, Lpd plays a key role in the regulation of cancer migration and appears to be a viable target to prevent metastasis.

In the present study, we investigated the therapeutic efficacy of *Ganoderma lucidum* extract (GLE) on BC cell motility via the Rac/Lpd pathway. *Ganoderma lucidum* is a traditional Chinese medicinal mushroom used for centuries to treat various diseases including cancer [19,20]. The effectiveness of *G. lucidum* anticancer properties has been linked to its bioactive compounds such as polysaccharides and triterpenes [21,22,23]. Moreover, numerous studies have focused on the efficacy of individual components rather than on the effects of the whole mushroom extract. The interaction between the different biologically active compounds within the whole mushroom extract (i.e., GLE), offers simultaneous effects that we and others have shown to selectively affect cancer cells [24,25]. Previous studies have shown that GLE suppresses BC cell growth and metastatic potential by inhibiting pro-invasive genes, transcriptional activators, and key signaling pathways, including urokinase-type plasminogen activator (uPA) and its receptor uPAR [21,26,27,28,29]. Moreover, our group has demonstrated that GLE displays anticancer effects in BC and inflammatory breast cancer models at doses that have no adverse effect on noncancerous cells [25]. We have also shown that GLE displays anti-tumor responses in mice and sensitizes cancer cells to treatment with conventional chemotherapies in vitro and in vivo [30,31]. Additionally, we have shown that GLE impairs breast cancer stem cells by targeting the STAT3 pathway [32].

Our hypothesis for this study is that GLE inhibits the formation of lamellipodia through the regulation of Rac/Lpd pathway leading to a reduction of BC cell migration and invasion. Our study is the first to show that GLE inhibits Lpd—a key regulator of lamellipodia formation—and the activity of Rac in cancer models.

## 2. Materials and Methods

### 2.1. Whole Mushroom Ganoderma Lucidum Extract (GLE)

A commercially available extract consisting of *Ganoderma lucidum* fruiting body and cracked spores, commercially known as ReishiMax GLp^®^, was purchased from Pharmanex^®^ Inc. (Provo, UT, USA). GLE is a mixture of 13.5% polysaccharides, 6% triterpenes, and 1% cracked spores. The extract is available in capsules, where the contents (500 mg) were dissolved in 10% sterile dimethyl sulfoxide (DMSO) (Sigma Aldrich, St. Louis, MO, USA) at a working stock of 100 mg/mL, then diluted to different working concentrations with media before being used as described in [31].

### 2.2. Cell Culture

The cell lines used were obtained from ATCC^®^ (Manasssas, VA, USA). The human breast cancer cell line MDA-MB-231 (ATCC^®^ HTB-26^TM^) was cultured in Dulbecco’s Modified Eagle’s Medium (DMEM) (Life Technologies, Rockville, MD, USA) supplemented with 10% fetal bovine serum (Corning, Corning, NY, USA) as in [27]. The human noncancerous mammary epithelial cell line MCF-10A (ATCC^®^ CRL-10317^TM^) was cultured in DMEM/Ham’s F12 (Life Technologies, Rockville, MD, USA) with 10% horse serum (Sigma Aldrich) as described in [25]. Culture media components were purchased from Life Technologies/Gibco (Rockville, MD, USA) [25]. Cells were tested regularly to ensure they were free from mycoplasma infection using the Mycoplasma Detection Kit (ASB-1310001, Nordic BioSite AB, Sweden). MDA-MB-231 and MCF-10A cell lines were genotyped for authenticity using the Short Tandem Repeat (STR) profile and interspecies contamination testing services from IDEXX BioResearch (Columbia, MO, USA).

### 2.3. Cell Viability

One-hundred thousand cells/well MDA-MB-231 and MCF-10A were seeded and cultured for 24 h at 37 °C in an atmosphere of 5% CO_2_. Then, the cells were treated in duplicate with vehicle (0.1% DMSO) or in 2-fold serial dilutions of GLE for 48 h. After the treatment period, the cells were fixed with cold methanol and the nuclei were stained with 0.4% propidium iodide (PI) (Sigma Aldrich). Fluorescence units were measured using a GloMax^®^ Microplate Reader (Promega, Madison, WI, USA). Cell viability was calculated as the percent of surviving cells after treatment relative to vehicle as in [30].

### 2.4. Wash Out Assays

MDA-MB-231 (1 × 10^5^) cells were treated with vehicle or GLE. After 48 h the treatment was removed and the cells were washed with phosphate buffered saline (1× PBS, pH 7.4) then incubated for another 72 h in fresh media. Then, the cell viability was determined as described before [30].

### 2.5. Wound Healing Assay

Twenty-thousand MDA-MB-231 cells/insert were cultured on two-well silicone inserts with a defined cell-free gap wound plate (Ibidi USA Inc., Madison, WI, USA) for 24 h. Then, the media was changed to starving media (DMEM) prior to the treatment with vehicle or GLE (0.96 mg/mL) for 24 h. The cells were fixed (4% paraformaldehyde and 0.1% Triton ×-100 in 1X PBS) and stained with the F-actin marker rhodamine phalloidin (Life Technologies) to visualize actin filaments (F-actin) and with 1 µg/mL of the nuclear marker DAPI (4′,6-diamidino-2-phenylindole) (Life Technologies) for nuclear staining. Cell migration was quantified by measuring the distance between the edges of the wound using ImageJ software V.1.50i (NIH, Bethesda, MD, USA) on a total of five micrographs at a magnification of 20X.The data was acquired from an inverted Olympus fluorescence microscope (Center Valley, PA, USA).

### 2.6. Invasion Assay

Cell invasion was measured using the BD BioCoat Matrigel^TM^ Invasion Assay (BD Biosciences, San José, CA, USA) [25]. Seventy-five-thousand quiescent cells/well were seeded in the top chambers, then treated with vehicle, 0.25 mg/mL, or 0.96 mg/mL GLE, and incubated at 37 °C to allow invasion toward 10% FBS medium (chemoattractant). After 72 h, cells on the upper membrane surface were removed with a cotton swab and cells attached to the bottom surface of the membrane were fixed and stained with propidium iodine [25]. Cells were quantified with ImageJ (NIH). Micrographs were obtained at a 40X magnification with an Olympus upright fluorescence microscope (Center Valley, PA, USA). Data were calculated as percent of invading cells after treatment relative to vehicle.

### 2.7. Rac Activity Assay

MDA-MB-231 cells were treated with vehicle or 0.96 mg/mL GLE for 24 h, then lysed on ice and the total proteins were extracted using the NP-40 lysis buffer as described by us [31]. Protein concentrations were determined with Precision Red^TM^ advanced protein measurement reagent (Cytoskeleton, Denver, CO, USA) according to the manufacturer’s instructions. Rac activity was measured by mixing cell lysates (2 mg/mL) with GTP-PAK-PBD beads (Cytoskeleton) in an end-over-end shaker for 2 h at 4 °C. The beads/lysate mixture was spun, supernatant was removed, and beads were washed in PBS. Then, beads were boiled in denaturing sample buffer for 5 min, and the supernatant was assessed by Western blot analysis using a primary antibody against Rac (1:1000, #2465) (Cell Signaling, Danvers, MA, USA).

### 2.8. Western Blot Analysis

MDA-MB-231 cells were treated with vehicle, 0.96 mg/mL, or 0.50 mg/mL GLE for 24 or 48 h, respectively. Cells were lysed on ice and proteins were extracted using NP-40 lysis buffer as described by us [31]. Total protein (30 μg) was resolved on SDS-PAGE and immunoblotted with the following rabbit monoclonal antibodies (Cell Signaling, Danvers, MA, USA) at a 1:1000 dilution; anti-Lamellipodin (#91138), anti-FAK-(#13009), anti-p-FAK (Tyr925)-(#3284), anti-WAVE-2 (#3659), anti-Cdc42 (#2466), anti-c-Myc (#9402), and anti-RhoC (#3430). Additionally, mouse monoclonal anti-β-actin (#A1978) (Sigma Aldrich) and polyclonal rabbit anti-Ena/VASP-rabbit (#ABT63) (Millipore, Burlington, MA, USA) antibodies were used at a 1:1000 dilution. Western blots were quantified using ImageJ (NIH).

### 2.9. Immunofluorescence

The fixing, permeabilization, and blocking solutions were prepared in 1X PBS. One-hundred-and-forty-thousand cells were seeded in coverslips and treated with GLE for 24 h. After the incubation, cells were fixed with 4% paraformaldehyde for 15 min, washed with 1X PBS, and permeabilized with 0.3% Triton X-100 for 15 min at room temperature. Cells were washed with 1X PBS and incubated with 5% normal goat serum (Vector Laboratories, Burlingame, CA, USA) for 1 h at room temperature. For labeling, fixed cells were incubated with a primary antibody against Lamellipodin (1:200, Cell Signaling) overnight at 4 °C, followed by three washes with 1X PBS. Coverslips were incubated with anti-Rabbit Alexa 488 (1:750, #44125, Cell Signaling) for 1 h at room temperature. After three washes with 1X PBS, cells were incubated for 1 min at room temperature with 1 µg/mL of DAPI (Life Technologies), followed by three washes with 1X PBS. Cells were mounted on slides with antifade medium (Life Technologies). The images were acquired by confocal microscopy with an Olympus BX60 microscope (Olympus; Tokyo, Japan) outfitted with the Olympus FV1000 confocal laser scanning system.

### 2.10. Statistical Analysis

Data are expressed as mean ± S.E.M. *p*-values were calculated using nonparametric *t*-tests with Mann–Whitney test or analysis of variance (ANOVA) with Bonferroni multiple comparison test. Statistical analyses were done using Graph Pad Prism v. 7.0 (San Diego, CA, USA), and differences were considered significant when *p* ≤ 0.05. Calculations of the IC_50_s were done with dose response curve fittings using the nonlinear regression parameter: dose–response–inhibition using Graph Pad Prism. Each experiment was performed in three or more independent biological replicates. The mean was calculated adding the independent replicates of each experiment and dividing the value by the total amount of independent replicates (e.g., *n* = 3).

## 3. Results

### 3.1. GLE Selectively Inhibits Cancer Cell Viability

**Figure 1:**
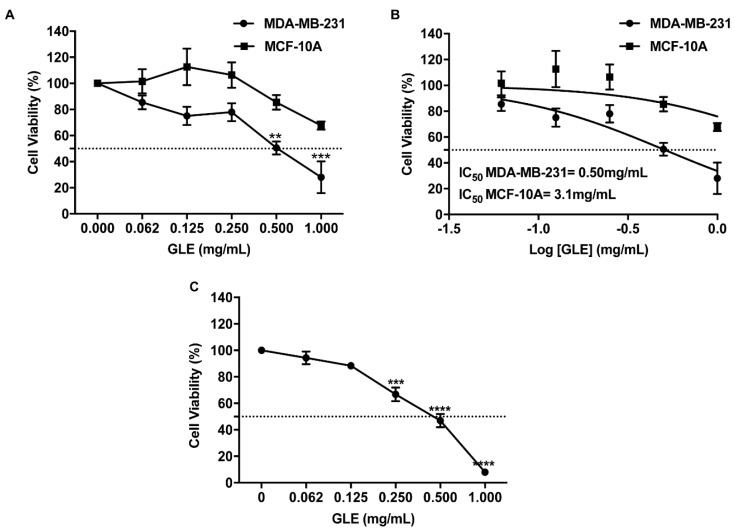
*Ganoderma lucidum* extract (GLE) decreases the viability of MDA-MB-231 breast cancer cells. (**A**,**B**) MDA-MB-231 (1 × 10^5^) and MCF-10A (1 × 10^5^) cells were treated with 0.062, 0.125, 0.250, 0.500, and 1.000 mg/mL of GLE for 48 h. Cell viability was calculated as described in materials and methods. IC_50_ was calculated from dose response curve fittings using nonlinear regression parameters. (**C**) MDA-MB-231 cells were treated with same concentrations of GLE as in (A) for 48 h. Treatment was removed after 48 h and the cells were incubated for an additional 72 h to assess recovery by a washout assay. Data are expressed as mean ± SEM. Experiments represent data obtained from three independent biological replicates. Statistically significant differences are shown at ** *p* < 0.01, *** *p* < 0.001, **** *p* < 0.0001 compared to vehicle.

To test the effectiveness of GLE on cell viability, we treated MDA-MB-231 and MCF-10A cells with 2-fold serial dilutions of GLE for 48 h. As shown in Figure 1A, GLE significantly decreased the viability of MDA-MB-231 cells after treating with 0.5 mg/mL (*p* < 0.01) or 0.96 mg/mL (*p* < 0.001) of GLE. Interestingly, GLE did not cause a significant effect on the viability of noncancerous MCF-10A cells (Figure 1A). The 48-h median inhibitory GLE concentration (IC_50_) was 0.50 mg/mL for MDA-MB-231 (Figure 1B); while for MCF-10A cells, the calculated IC_50_ was 6-fold higher when compared to MDA-MB-231 BC cells. To assess if MDA-MB-231 cells recover after treatment a washout assay was performed using the same concentrations as the dose response used for the cell viability assays. After 48 h of treatment, and a subsequent incubation without treatment for 72 h, our results show that MDA-MB-231 cells do not recover from the GLE treatment (Figure 1C). These results suggest that GLE is a selective anticancer treatment and that the effect is sustained for an extended period of time.

### 3.2. GLE inhibits Cancer Cell Migration and Invasion

**Figure 2:**
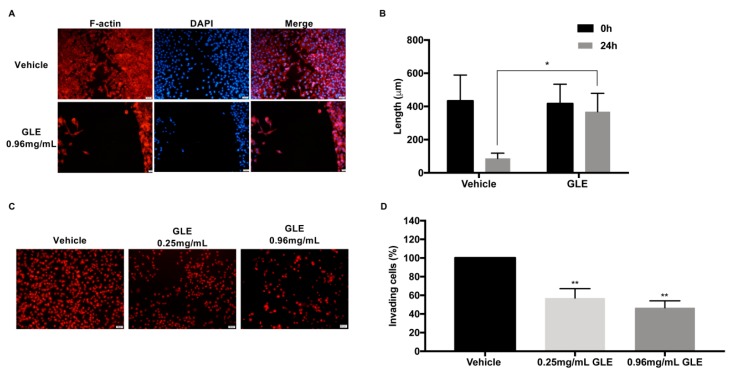
GLE decreases the migration and invasion of MDA-MB-231 breast cancer cells. (**A**,**B**) MDA-MB-231 (2 × 10^4^) cells were seeded onto two-well silicone insert with a defined cell-free gap wound healing assay plates and grown in 5% FBS media before treatment with vehicle (0.1% DMSO) or GLE (0.96 mg/mL) for 24 h. Cells were fixed and stained with rhodamine phalloidin and DAPI as described in materials and methods. We quantified 36 micrographs that were taken at a magnification of 20X. The migratory effect of the cells was quantified by measuring the distance between the edges of the wound with ImageJ V.1.50i. (**C**,**D**) Quiescent MDA-MB-231 (7.5 × 10^4^) cells were seeded in Transwell^®^ chambers and treated with vehicle, 0.25 mg/mL, and 0.96 mg/mL GLE for 24 h. Cells were fixed and PI-stained. The invasion ability of the cells was quantified by counting the number of cells that invaded through the extracellular matrix. Approximately, 36 micrographs were taken at a magnification of 20X and quantified using ImageJ. Data are expressed as mean ± SEM. Experiments represent data obtained from three independent biological replicates. Statistically significant differences are shown at * *p* < 0.05, ** *p* < 0.01 compared to vehicle. Scale bars = 20 μm.

Migration and invasion are critical steps for tumor progression and metastasis. Thus, to determine the inhibitory effect of GLE on migration, a wound healing assay was performed. MDA-MB-231 cells were treated with vehicle or the 24 h IC_50_ (0.96 mg/mL) of GLE. This IC_50_ concentration is in accordance to values previously reported by us and others [26,27,28,32]. Cells were treated, fixed, and stained with rhodamine phalloidin to visualize F-actin and DAPI to visualize the nuclei of migrating cells as described in [33]. As shown in Figure 2A,B, MDA-MB-231 cells treated with GLE show a significant reduction in wound closure (*p* < 0.05) compared to vehicle treated cells after 24 h of GLE treatment. Next, we assessed the effect of GLE on cell invasion by means of Matrigel-coated invasion chambers. Quiescent cells were treated with vehicle, a nonlethal dose of 0.25 mg/mL or 0.96 mg/mL of GLE for 24 h. The results show that both concentrations of GLE significantly decrease the invasion capacity of MDA-MB-231 cells (*p* < 0.01) (Figure 2C,D). Results suggest that GLE inhibits motility of BC cells.

### 3.3. GLE Affects Rac Activity

**Figure 3:**
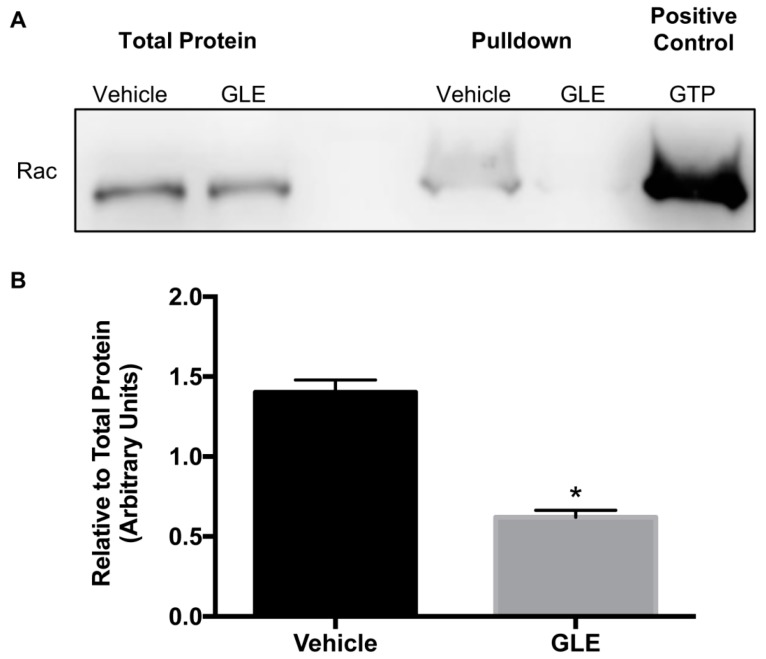
GLE decreases Rac activity in MDA-MB-231 breast cancer cells. (**A**) MDA-MB-231 cells were treated with vehicle or GLE (0.96 mg/mL) for 24 h. Total lysates were obtained and incubated with PAK-PDB beads to precipitate active Rac by a pulldown activation assay. The total protein lysates and pulldowns were analyzed by Western blot. GTP was used as a positive control to test activity of the beads. (**B**) Densitometry analysis of western blot bands. Quantification was done using integrated density units and Rac activity was calculated relative to total protein expression. Data are expressed as mean ± SEM. Experiments represent data obtained from three independent biological replicates. Statistically significant difference is shown at * *p* < 0.05 compared to vehicle.

Rac is a key molecular switch that promotes cancer cell migration/invasion and survival [10]. Rac is activated by GDP/GTP exchange regulated by GEFs, and an increase in Rac-GTP expression or activity has been linked with cancer progression. Likewise, previous studies have demonstrated that Rac potentially activates cell motility in breast tumor cells [8]. Therefore, activity of GTP-loaded Rac was measured by a pulldown activation assay. Cell lysates were incubated with p21 activated kinase-p21 binding domain (PAK-PBD) beads to precipitate active Rac. Total protein and pulldowns were tested by immunoblotting. As shown in Figure 3A there is no change in Rac total protein. However, results demonstrate a decreased Rac activity on GLE treated cells. Figure 3B shows that there is a significant reduction of Rac activity in MDA-MB-231 cells upon treatment with GLE compared to vehicle. These results suggest that GLE has an inhibitory effect on Rac activity thus inducing a reduction on cancer cell migration and invasion.

### 3.4. GLE Decreases Expression of Proteins Involved in Cell Migration

**Figure 4:**
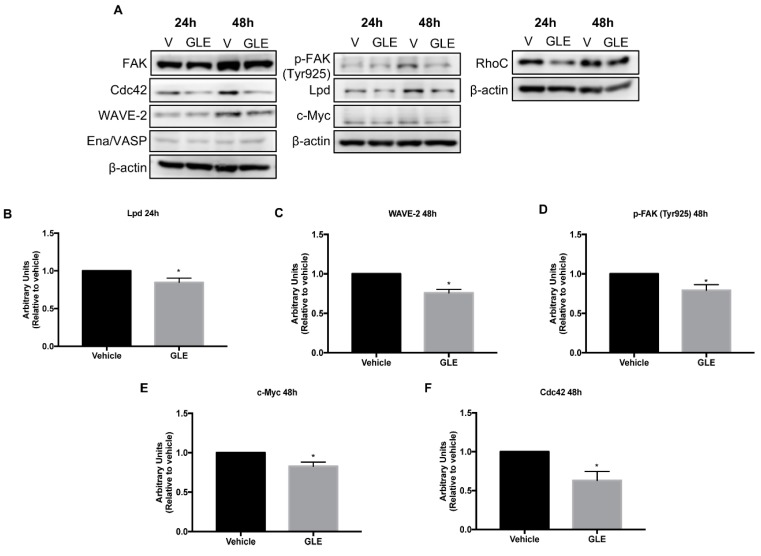
GLE modulates the expression of proteins involved in cell migration in MDA-MB-231 breast cancer cells. (**A**) MDA-MB-231 cells were grown in 5% FBS media for 24 h prior to treatment with vehicle (0.1% DMSO) or GLE (0.96 mg/mL, 0.50 mg/mL) for 24 and 48 h, respectively, before lysis. Equal amount of protein (30 µg) from each sample was used for western blot analysis. β-actin was used as a loading control. (**B**–**F**) Densitometry analysis of Western blot bands. Data are expressed as mean ± SEM. Experiments represent data obtained from three independent biological replicates. Statistically significant difference is shown at * *p* < 0.05 compared to vehicle.

Because GLE reduced cancer cell migration, invasion, and Rac activity, all of which have a major role in the development of membrane protrusions, we sought to investigate the effects of GLE on Rac signaling which leads to lamellipodia formation. Therefore, we assessed the expression of proteins involved in the process of cell migration and lamellipodia formation upon GLE treatment in breast cancer cells (Figure 4A). MDA-MB-231 cells were treated with vehicle, 0.96 mg/mL or 0.5 mg/mL of GLE for 24 and 48 h, respectively. Western blot analysis showed that GLE significantly reduces (*p* < 0.05) the expression of Lpd in MDA-MB-231 cells (Figure 4B). Lpd binds to active Rac promoting the interaction with the Scar/WAVE complex at the leading edge of the cells and regulating Scar/WAVE-Arp2/3 activity and thus lamellipodium formation and cell migration [14]. WAVE-2, a member of the Scar/WAVE complex, was also significantly inhibited (*p* < 0.05) upon GLE treatment (Figure 4C). Interestingly, Ena/VASP an actin regulator controlled by Lpd did not display a difference in expression in GLE treated cells (Figure 4A). Next, considering FAK plays an important role on focal adhesion formation and cell migration we studied the expression of focal adhesion kinase FAK and p-FAK (Tyr925). The specific phosphorylation of p-FAK in the Tyr925 coordinates the focal adhesion disassembly or formation of cell edge protrusions, which are important processes in cell migration [34]. Treatment with GLE did not alter the levels of total FAK but decreased significantly the expression of p-FAK (Tyr925) (Figure 4D). FAK signaling mediates tumorigenesis through activation of c-Myc and studies showed that this functional link is due to a transcriptional activation of c-Myc by FAK [35]. Our results demonstrate a significant decrease (*p* < 0.05) of c-Myc expression after GLE treatment (Figure 4E) in BC cells. Additionally, we tested the expression of other Rho-family GTPases, such as Cdc42 and RhoC that are involved in cell migration and stimulate the formation of filopodia and stress fibers, respectively. Our results displayed a significant downregulation in expression (*p* < 0.05) of Cdc42 in GLE treated cells (Figure 4F), while no effect was detected in RhoC expression. Together, these results suggest that GLE attenuates the expression of key proteins involved in Rac signaling, which mediate cell migration, invasion processes, and lamellipodia formation in BC cells.

### 3.5. GLE Affects Lamellipodia Formation in MDA-MB-231 Cells

**Figure 5:**
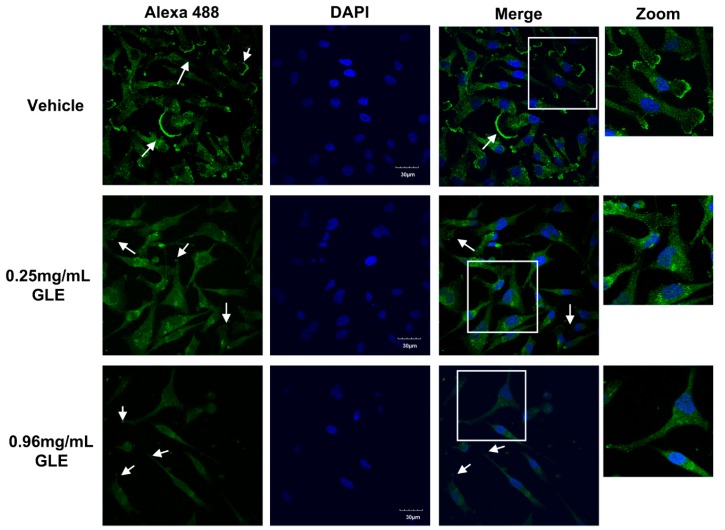
GLE inhibits Lpd expression in MDA-MB-231 breast cancer cells. MDA-MB-231 (1.4 × 10^4^) cells were grown in 5% FBS media for 24 h prior to treatment with vehicle or GLE (0.25 mg/mL, 0.96 mg/mL) for 24 h. Cells were fixed and stained with DAPI (blue) and anti-Lamellipodin (green) per manufacturer’s instructions. White arrows show lamellipodia. Experiments represent data obtained from three independent biological replicates. Zoom in each image is shown in white squares, and each share equal dimensions. Scale bars = 30 μm.

Rac regulates actin polymerization and cell surface extensions through effectors such as lamellipodin (Lpd). Thus, we assessed whether GLE attenuates the formation of lamellipodia in MDA-MB-231 cells. BC cells were treated with vehicle, 0.25 mg/mL and 0.96 mg/mL of GLE for 24 h. Immunofluorescent staining showed a markedly expression of Lpd localized in the ruffled edges of vehicle treated cells (Figure 5). However, there was a clear reduction in Lpd expression in cells treated with GLE compared to vehicle. A remarkable change in cell morphology was observed in GLE treated cells, where nuclei appeared smaller, the cells show reduced lamellipodium formation and no membrane ruffles were detected. These data suggest that GLE inhibits Lpd expression and lamellipodia formation in BC cells.

## 4. Discussion

In this work we demonstrated that GLE decreases cancer cell viability in a dose-dependent manner in the highly aggressive and invasive triple-negative BC cell line MDA-MB-231. These results agree with previous findings from our group where we established that GLE dose-dependently reduces the cell viability of other highly invasive BC cells (MDA-MB-468, MDA-MB-435, SUM-102, and SUM-149) in a dose-dependent manner by various mechanisms including via apoptosis induction [25,31,32]. These results are sustained by the work of Wu and collaborators who have reported that MDA-MB-231 cell viability is compromised by treatment with Ganoderiol-A, a bioactive triterpenoid derived from *G. lucidum* [36]. Furthermore, in the current work we report that GLE did not affect the viability of noncancerous mammary epithelial cells 48h after treatment, which correlates with our previous findings where this cell line was not inhibited by GLE treatment at a shorter time point [25]. Here, we showed that MDA-MB-231 BC cells did not recover from GLE treatment which support our previous findings establishing that SUM-149 and SUM-102 BC cells did not recover from GLE [30]. Our results suggest that GLE is selective upon BC cells and indicate a long-term effectiveness supporting its potential as an anticancer treatment.

Cancer cells participate in cell migration and invasion processes to promote tumor progression and metastasis [37]. Our data showed that GLE affected BC cell migration and their invasiveness capacity in accordance with our previous work [25,30,31]. One of the major effectors involved in cell migration and invasion is the Rho family GTPases which regulates the organization of the actin cytoskeleton to mediate the formation of filopodia, lamellipodia, and stress fibers [38]. Among them, Rac, which is related to lamellipodia formation, has been implicated in tumorigenesis and metastasis by having a crucial role in cellular functions of cancer cell motility and survival. The deregulation of Rac signaling has been linked to the enhancement of upstream signals from tyrosine kinase receptors, PI3K, GEFs or reduced inactivation by GTPase activating proteins. For example, phosphatidylinositol (3,4,5)-trisphosphate [PtdIns (3,4,5) P3] (PIP_3_) is the product of the activation of PI3K through phosphatidylinositol (4,5)-bisphosphate (PIP_2_) phosphorylation. PIP_3_ is also an upstream effector of Rac GEFs that regulates its activity though a feedback loop with PI3K thus promoting F-actin polymerization [39]. PI3K activation also recruits Lpd to the plasma membrane through PIP_3_ [16]. Interestingly, we and others have shown that GLE inhibits the PI3K/Akt signaling pathway [26,31], which is downstream of PI3K, suggesting that GLE has a modulating effect on PI3K activation. Thus, GLE’s effect on the PI3K/Akt pathway could reduce the availability of Rac GEFs, thus suppressing Rac activity and impairing Lpd recruitment. A study where Rac inhibitor EHT186 was tested showed that inhibition of Rac activity promoted the loss of guanine nucleotide association, locked Rac in an inactive conformation, and inhibited the GTPase activity and the engagement of downstream effectors in BC cells [40]. Rac has been found to be overexpressed in BC tissues and has been reported as a key molecular switch that promotes BC migration and invasion [8]. Herein, we tested the activity of Rac (Rac-GTP) demonstrating that whole mushroom GLE significantly reduces Rac activity with no effect on total Rac protein expression. These results contrast with the work from Wu et al. who demonstrated a downregulation in total Rac expression by treatment with the specific derived compound Ganoderiol A-enriched extract (GAEE), a derived *G. lucidum* compound [36]. Nevertheless, our results suggest that downregulation of Rac activity by GLE decreased BC cell migration and invasion supporting its potential as an antimigratory and anti-invasion treatment.

Since GLE significantly reduces Rac activity, we further focused on the elucidation of GLE effects on Rac signaling. One of the essential roles of Rac is the regulation of the cytoskeleton organization by promoting the formation of lamellipodia [8]. The Rac effector, Lpd is involved in lamellipodial dynamics and localizes at the tips of lamellipodia and filopodia. Lpd directly interacts with active Rac through the association of Ras and pleckstrin homology domains, which in turn regulates the interaction with the Scar/WAVE complex [14]. The WAVE complex is composed of various subunits including WAVE-2 which has been found in high levels in BC patients [41]. WAVE-2 has a role in lamellipodia turnover and Rac-GTP is required for its activation [16]. We show that GLE significantly reduced the expression of Lpd and WAVE-2 in BC cells. Lpd also interacts with Ena/VASP promoting the formation of lamellipodia by regulating the length and branching of actin filaments [15]. Ena/VASP proteins control actin filament length by preventing the capping of barbed ends and by recruiting profilin–actin to the growing end of actin filaments [42]. Our data shows that GLE had no effect on Ena/VASP expression. The difference in Lpd-Ena/VASP and Lpd-Scar/WAVE regulation allows Lpd to balance actin cytoskeletal dynamics via Ena/VASP-mediated actin filament elongation and Scar/WAVE-mediated nucleation/branching [18]. These results suggest that GLE mostly affects the Rac/Lpd/WAVE signaling which in turns results in branching retarding, thus reducing lamellipodia formation.

Moreover, as it has been shown in previous work, Lpd promotes actin remodeling by the interaction with Ena/VASP which localizes to focal adhesions [15]. One of the proteins regulating focal adhesion formation is FAK which has an important role in the cross-talk between focal adhesions and cell protrusions [34]. We showed that GLE treatment causes Lpd downregulation and subsequent p-FAK (Tyr925) inhibition with no changes in total FAK. The phosphorylation in Tyr925 plays an important role in cell edge protrusions [34]. Therefore, reduction in its expression may lead to the decrease of lamellipodia formation in BC cells. Another protein affected by GLE treatment is c-Myc, which is a downstream effector of FAK. c-Myc has been shown to be regulated by integrin alpha-V (ITGAV) through the activation of the FAK-p38-p90RSK signaling axis that promotes 3D tumor invasion [43]. Here we demonstrated GLE’s decreasing effect on Rac activity and reduced c-Myc expression. These results suggest that GLE not only affects proteins that promote the turnover of lamellipodia formation but also modulates the expression of focal adhesion proteins and downstream effectors of FAK.

Other Rho family GTPases, such as Cdc42 and Rho, play an important role in the cell migration process by participating in the formation of filopodia and stress fibers. Cdc42 also plays a role in lamellipodia, and has been reported to regulate directionality of movement and localization of lamellipodial activity to the leading edge [44]. Previous studies have demonstrated that overexpression of Cdc42 in BC cells promotes tumorigenesis by altering Rho GTPase and MAPK signaling [45]. We showed that Cdc42 was significantly inhibited upon treatment with GLE. GLE showed no effect in RhoC expression. Altogether, our findings suggest a potential and novel mechanism for GLE to disrupt lamellipodia formation, and ultimately decrease cell migration.

We examined the effect of GLE in lamellipodia formation to elucidate whether GLE affected the formation of these membrane protrusions. Our results showed that vehicle treated cells display a strong Lpd membrane localization on the cell edges with remarkable ruffle formation while, there was a visible reduction in Lpd and lamellipodia formation in BC cells. Data from other groups have showed that downregulation of Lpd retards the lamellipodia formation in BC cells [18]. Our data suggest that treatment with GLE disrupts the accumulation of Lpd in BC cell protrusions.

In summary, we demonstrated for the first time that GLE suppresses the activity of Rac affecting downstream signaling pathway molecules that are important in lamellipodia structure formation. These events result in cell migration and invasion reduction of BC cells. One limitation of the current study is that these are preliminary *in vitro* results that will need further testing in *in vivo* models to fully understand and elucidate GLE’s full therapeutic potential. Nevertheless, our novel findings lay the foundation for further preclinical studies to investigate the role of GLE in the regulation of the Rac/Lpd pathway, as well as in the progression and metastasis of BC cancer.
